# Pushing the Limits of Spatial Assay Resolution for Paper-Based Microfluidics Using Low-Cost and High-Throughput Pen Plotter Approach

**DOI:** 10.3390/mi11060611

**Published:** 2020-06-24

**Authors:** Reza Amin, Fariba Ghaderinezhad, Caleb Bridge, Mikail Temirel, Scott Jones, Panteha Toloueinia, Savas Tasoglu

**Affiliations:** 1 Department of Mechanical Engineering, University of Connecticut, Storrs, CT 06269, USA; reza.amin@uconn.edu (R.A.); fariba.ghaderinezhad@uconn.edu (F.G.); caleb.bridge@uconn.edu (C.B.); scott.jones@uconn.edu (S.J.); 2 UConn School of Engineering Professional Education, University of Connecticut, Storrs, CT 06269, USA; 3 Department of Biomedical Engineering, University of Connecticut, Storrs, CT 06269, USA; mikail.temirel@uconn.edu; 4 Institute of Materials Science (IMS), University of Connecticut, 97 North Eagleville Road, Storrs, CT 06269, USA; panteha.toloueinia@uconn.edu; 5 Department of Mechanical Engineering, Koç University, Sariyer, Istanbul 34450, Turkey; 6 Koç University Arçelik Research Center for Creative Industries (KUAR), Koç University, Sariyer, Istanbul 34450, Turkey; 7 Boğaziçi Institute of Biomedical Engineering, Boğaziçi University, Çengelköy, Istanbul 34684, Turkey; 8 Koç University Research Center for Translational Medicine, Koç University, Sariyer, Istanbul 34450, Turkey

**Keywords:** high-resolution, miniaturized paper-based assay, multiplex assays, medical diagnostics, microfluidics, colorimetric analysis

## Abstract

To transform from reactive to proactive healthcare, there is an increasing need for low-cost and portable assays to continuously perform health measurements. The paper-based analytical devices could be a potential fit for this need. To miniaturize the multiplex paper-based microfluidic analytical devices and minimize reagent use, a fabrication method with high resolution along with low fabrication cost should be developed. Here, we present an approach that uses a desktop pen plotter and a high-resolution technical pen for plotting high-resolution patterns to fabricate miniaturized paper-based microfluidic devices with hundreds of detection zones to conduct different assays. In order to create a functional multiplex paper-based analytical device, the hydrophobic solution is patterned on the cellulose paper and the reagents are deposited in the patterned detection zones using the technical pens. We demonstrated the effect of paper substrate thickness on the resolution of patterns by investigating the resolution of patterns on a chromatography paper with altered effective thickness. As the characteristics of the cellulose paper substrate such as thickness, resolution, and homogeneity of pore structure affect the obtained patterning resolution, we used regenerated cellulose paper to fabricate detection zones with a diameter as small as 0.8 mm. Moreover, in order to fabricate a miniaturized multiplex paper-based device, we optimized packing of the detection zones. We also showed the capability of the presented method for fabrication of 3D paper-based microfluidic devices with hundreds of detection zones for conducting colorimetric assays.

## 1. Introduction

The increasing costs of healthcare services and health insurance premiums drive the need to transition from reactive and hospital-centered care to proactive healthcare management and wellness. This shift from a reactive to a more proactive approach, encompassing preventive, evidence-based, and person-centered care, improves the quality of care and decreases the costs of healthcare [1]. To meet this much-needed transformation, low-cost and compact technologies should be developed to continuously perform health measurements [2]. Ease of accessibility to low-cost assays for continuous health monitoring is essential to improve patients’ well-being.

To provide a low-cost and portable assay for health measurements, miniaturizing paper-based assays will play an important role. In addition to reducing the sample volumes needed for assays, miniaturization will also decrease the consumption of reagents and quantity of waste produced [3]. A further benefit of miniaturization is fast and efficient chemical reactions [4,5]. Moreover, miniaturized portable medical diagnostic assays with low power consumption, stand-alone functionalities, and less need of clinical expertise are more important for the people in developing countries who do not have direct access to medical laboratories with basic diagnostic and analytical facilities [6,7]. To be able to decrease the size of the paper-based assays and miniaturize them, an appropriate high-resolution fabrication method should be found to meet all other needs of high-throughput fabrication such as being low-cost, simple, and easy.

Several paper-based microfluidic device fabrication methods have been reported in recent studies [8,9,10,11,12,13,14,15,16,17,18,19,20,21,22,23,24,25,26,27,28,29,30], all of which utilize the hydrophilic features of the paper substrate and some form of hydrophobic ink patterned as a barrier. The fabrication techniques can be broken down into analog and digital fabrication methods. Analog methods, which require the use of a pre-fabricated mask or plate, include photolithography, screen printing, flexographic printing, stamping, lacquer spraying, and plasma treatment (Table S1). Digital methods, as opposed to analog methods, allow for near-instantaneous changes to the pattern; these methods include wax printing, inkjet printing, paper cutting, inkjet etching, laser etching, and pen plotting (Table S2). For low-cost, point-of-care diagnostic devices, a method that could dynamically alter the fabrication pattern and reagent deposition through digital means has a significant advantage. We have compared both analog and digital fabrication methods based on several comparison metrics including resolution, throughput, required equipment, and cost as well as the limitations and advantages (Tables S1 and S2). Among those approaches, the methods that are appropriate for mass-scale fabrication are wax printing [19,26,27], inkjet printing [5,8,21], screen printing [18], flexographic printing [12], and pen plotting [29,30] with the reported channel resolution of 228 ± 30 µm [10], 272 ± 19 µm [12], 671 ± 50 µm [2], 500 ± 30 µm [3], and 391 ± 68 μm [30], respectively. The advantages of the pen plotting method are its simple workflow and affordability. Moreover, unlike most of the other methods suitable for mass-scale fabrication, this method can deposit the reagents of biochemical assays into the detection zones, helping to make the process completely automated. The pen plotting method offers these advantages while preserving the main features of other mass-scale fabrication techniques, i.e., simple and low-cost fabrication. For low-cost, point-of-care diagnostic devices [31,32,33,34,35,36], the ability to dynamically alter the pattern through digital means is a significant advantage as well.

**Figure 1:**
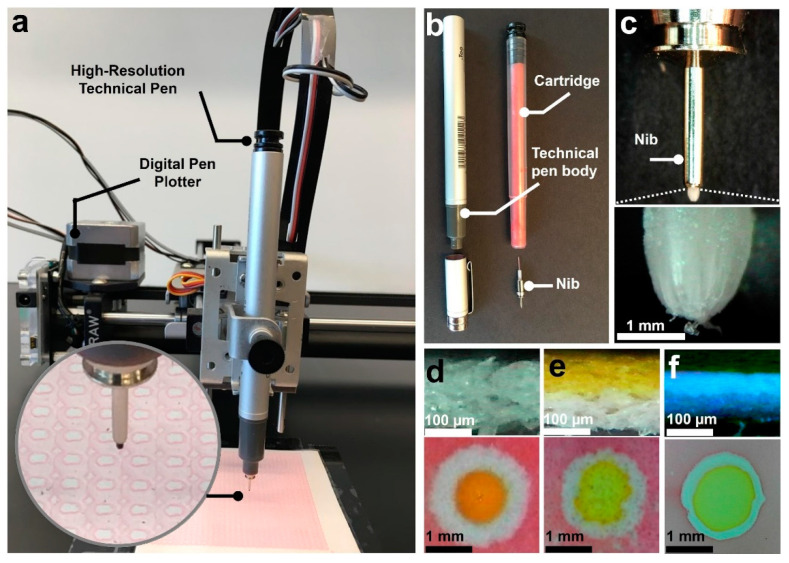
High-resolution fabrication of multiplex paper-based microfluidic devices using a pen plotter machine and high-resolution technical pens. (**a**) Desktop pen plotter with a high-resolution technical pen. (**b**) High-resolution technical pen with 0.1 mm plastic tip used for patterning hydrophobic solution to create detection zones. (**c**) Technical pen nib with 0.1 mm plastic tip used for patterning hydrophobic solution on paper substrate. (**d**,**e**) Cross-section of chromatography paper (**d**), chromatography paper after being coated with hydrophobic solution using the wide marker (**e**), regenerated paper (**f**), and corresponding circular pattern (2 mm diameter) on those paper substrates. The yellow food dye is applied to the hydrophilic side of paper to distinguish it from hydrophobic side.

In response to the need of finding a low-cost and high-throughput fabrication method to produce miniaturized paper-based assays [37,38,39], we have developed an approach using a desktop pen plotter and technical pens (Figure 1). A commercially available water-based hydrophobic solution with an added colorant for visualization of the printed pattern was used for plotting hydrophobic barriers. In addition to the method used for patterning, characteristics of paper substrate such as the thickness, porosity, and orientation of fibers are also important in determining the resolution of a fabrication method [40]. To demonstrate the effect of thickness on the resolution of patterns, we coated different thicknesses of the hydrophobic solution on Whatman grade 1 paper to get papers with different effective thicknesses and then investigated the resolution of plotted detection zones on those papers. Moreover, we tested Whatman regenerated paper with higher resolution and homogeneity of pore structure to investigate the highest achievable resolution using the presented fabrication method. Additionally, we performed an optimization to investigate how tightly we can pack the plotted patterns to fabricate the smallest possible microfluidic devices. Finally, to demonstrate the capability of the presented method for fabrication of paper-based diagnostic devices with multiple detection zones, we conducted hundreds of pH colorimetric assays on a single device.

## 2. Materials and Methods

### 2.1. Materials

*Hardware setups:* Desktop pen plotter, AxiDraw (Evil Made Scientist Laboratories, Sunnyvale, CA, USA); wide Copic marker (Imagination International Inc., Eugene, OR, USA); broad calligraphy wide nib for wide Copic marker (Imagination International Inc., Eugene, OR, USA); MakerBot Replicator 3D printer (MakerBot Industries, New York City, NY, USA); Form2 3D printer (Formlabs Inc, Somerville, MA, USA); Copic Multiliner SP technical pen, 0.1, 0.05, and 0.03 mm diameter nib sizes (Imagination International Inc., Eugene, OR, USA); benchtop laminator, GBC Inspire (Swingline, Lake Zurich, IL, USA); pH meter (35624-23) (Cole-Parmer Instrument Company, Vernon Hills, IL, USA); MM-840 EFLEX digital microscope (Carson Optical, Ronkonkoma, NY, USA); and CO2 laser cutter (Universal Laser Systems, Inc., Scottsdale, AZ, USA).

*Materials and reagents:* Super hydrophobic water repelling treatment, NeverWet auto interior (NeverWet LLC, Lancaster, PA, USA); chromatography paper, Whatman No. 1 (GE Healthcare Life Sciences, Bloomington, IL, USA); Whatman regenerated paper, pore size 1 μm (GE Healthcare Life Sciences, Bloomington, IL, USA); Fusion 5 (8151-9915) (GE Healthcare Life Sciences, Bloomington, IL, USA); hot-laminating layer, GBC EZUse thermal laminating pouches (Swingline, Lake Zurich, IL, USA); double-sided tape (Chica and Jo, Lake Zurich, IL, USA); cellulose powder (310697, Sigma-Aldrich, St. Louis, MO, USA); methyl red sodium salt (114502) and bromothymol blue (114413) (Sigma-Aldrich, St. Louis, MO, USA); buffer solution pH 4.01 (00654-00) and 10.01 (00654-08) (Cole-Parmer Instrument Company, Vernon Hills, IL, USA); and ethanol (A405F) (Thermo Fisher Scientific Inc., Waltham, MA, USA).

### 2.2. Control of the Pattern Depth to Improve the Resolution

**Figure 2:**
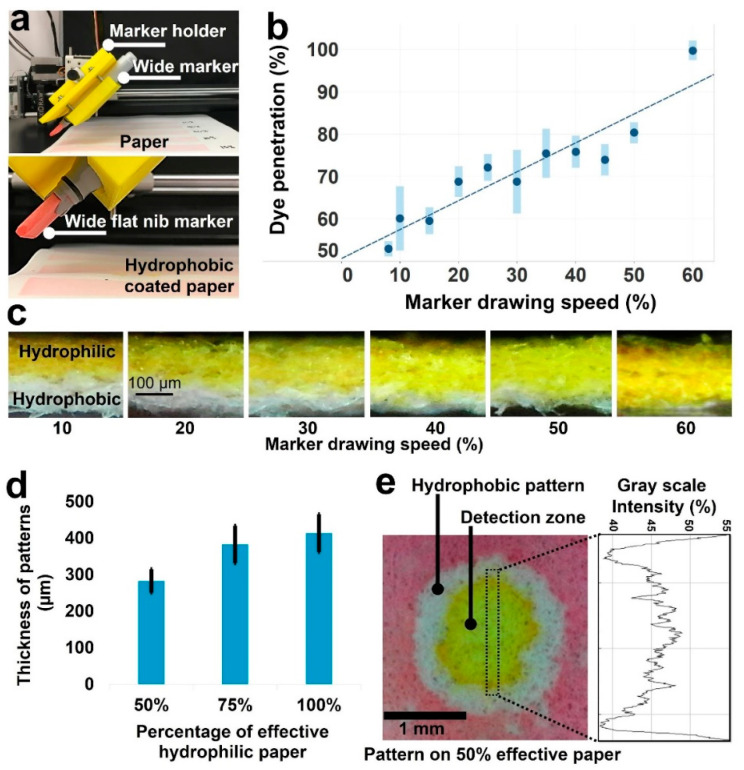
Evaluation of decreasing effective paper thickness on the resolution of patterns. (**a**) The custom-designed wide marker holder connected to the pen plotter to deposit a layer of hydrophobic ink on the chromatography paper. (**b**) Graph of percentage of food dye penetration through the thickness of paper (indicating the effective hydrophilic thickness of paper) versus the plotting speed percentage used to plot hydrophobic solution on the backside of chromatography paper. (**c**) Images of chromatography paper cross-sections after being coated with hydrophobic solution using the wide marker plotted for various speeds. The yellow food dye is applied to the hydrophilic side of paper to distinguish it from the hydrophobic side of paper. (**d**) Graph of line thickness of patterns on the chromatography paper with 50%, 75%, and 100% effective thickness (*N* = 6 and the error bar shows the standard deviation). (**e**) Image of a circular pattern (2 mm diameter) on the chromatography paper with 50% effective thickness and grayscale intensity analysis of food dye in detection area.

**Figure 3:**
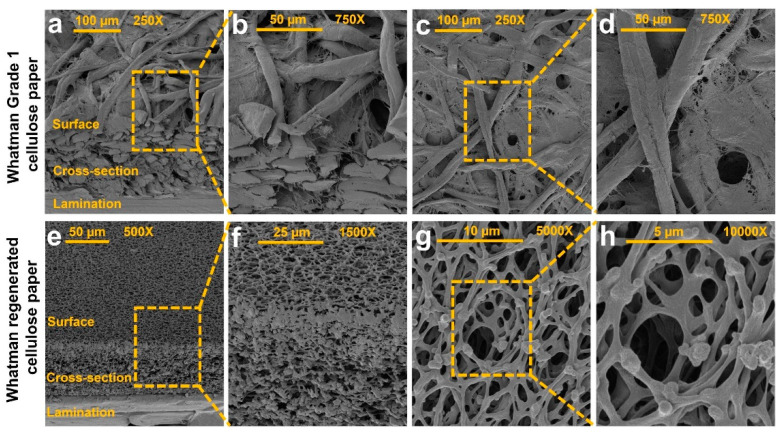
Physical characterization of Whatman grade 1 and regenerated cellulose paper. (**a**) SEM image with 250× magnification and (**b**) 750× magnification of the laminated Whatman grade 1 cellulose paper, showing the 45° view of surface and cross-section. (**c**) SEM image with 250× magnification and (**d**) 750× magnification of the Whatman grade 1 cellulose paper surface. (**e**) SEM image with 500× magnification and (**f**) 1500× magnification of the laminated Whatman regenerated cellulose paper, showing the 45° view of surface and cross-section. (**g**) SEM image with 5000× magnification and (**h**) 10,000× magnification of the Whatman regenerated cellulose paper surface.

When depositing hydrophobic patterns on the paper, there is a direct relationship between the paper thickness and the time needed for the ink to fully wick through the thickness of the paper. Consequently, thinner paper allows for pen plots to be made quicker, and a higher resolution to be achieved. For a circle-shaped pattern, an increase in resolution can reduce the inner radius of the circle, and therefore maximize the number of detection zones per unit of area. Using Whatman No. 1 paper, we reduced the paper’s effective thickness by evenly coating one side with a hydrophobic solution. To automate this process, we designed and 3D printed a holder for a wide Copic marker with 3 cm nib size that was retrofitted to an AxiDraw pen plotter (Figure 1a). For varying speeds, the mechanism evenly coated one side of the paper, which was then baked at 150 °C for 30 min to cure. To analyze the experimental results, we drew patterns on the opposite side of the paper and imaged the cross-section using a digital microscope (Figure 2a–c). With the images, we used the ImageJ image processing program to make measurements of pattern depth and reported the average and standard deviation for each corresponding coating speed. For each pen speed, five circles were plotted and measurements were taken every 90° along the circle (Figure 2d,e). Furthermore, as the physical characteristics of the cellulose paper substrate such as thickness, resolution, and homogeneity of pore structure affect the obtained patterning resolution, we characterized the physical characteristics of both Whatman grade 1 and regenerated paper using scanning electron microscopy (Figure 3).

### 2.3. The Effect of Nib Size on Resolution

To determine the smallest possible resolution of the Copic Multiliner SP pen, we first tested the maximum speed at which each pen could plot while still allowing the ink to fully penetrate the paper, creating a completely hydrophobic barrier. The three nib sizes we tested were 0.03 mm, 0.05 mm, and 0.1 mm diameters. To find the maximum speed of each nib, 13 rows of 2-mm circles were plotted on laminated Whatman regenerated paper (pore size of 1 μm) using the AxiDraw pen plotter. The Axidraw’s reported *X* and *Y* resolution and reproducibility by the manufacturer are 12.5 µm and 100 µm, respectively. The first row was then plotted at 1% speed, and then each proceeding row was plotted 1% faster, up to 13% speed. We then clamped the paper between glass slides and cured it in an oven for 30 min at 150 °C to allow the ink to cure, transitioning it to be super hydrophobic. Once cured, we tested the water resistance of each circle by adding a droplet of yellow food dye to the center of the circle. The highest speed that did not allow the yellow dye solution to escape a single circle was then chosen to be the ideal speed.

**Figure 4:**
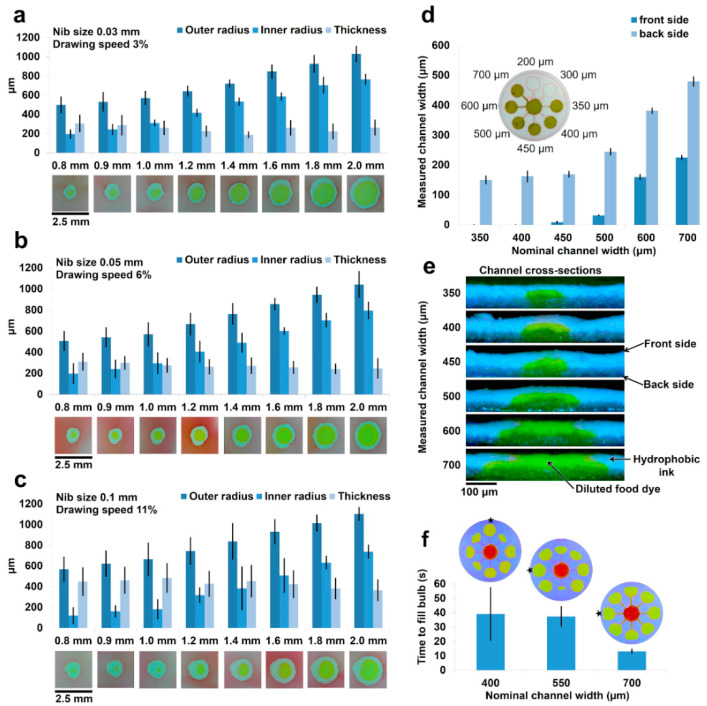
Characterization of high-resolution fabrication of detection zones and channels. (**a**) The resolution of 0.03 mm fiber technical pen on regenerated paper with optimal 3% drawing speed (*N* = 9 and the error bar shows the standard deviation); (**b**) the resolution of 0.05 mm fiber technical pen on regenerated paper with optimal 6% drawing speed (*N* = 9 and the error bar shows the standard deviation); (**c**) the resolution of 0.1 mm fiber technical pen on regenerated paper with optimal 11% drawing speed (*N* = 9 and the error bar shows the standard deviation). The results measured using MATLAB script at 8 angles around each reaction area across reaction areas. (**d**) The graph of the measured microfluidic flow channel width versus the plotted channel width (*N* = 8 and the error bar shows the standard deviation). The channels are plotted using the 0.1 fiber technical pen with 11% drawing speed and the channel widths are measured in ten equidistant locations using ImageJ software across three test repeats. (**e**) The cross-sectional image taken of each channel. The channels are filled with yellow food dye to clarify channel vs. barrier. (**f**) The average time to fill bulb for each corresponding equal channel test (*N* = 8 and the error bar shows the standard deviation). The color-altered images above the chart display the flow state as the first bulb was completely filled (star marks first bulb to fill).

We then used the ideal speed from each speed test to plot the corresponding resolution test for each nib size. We plotted 8 rows of circles, each row with a different diameter, on the laminated regenerated paper. The rows had radii of 2, 1.8, 1.6, 1.4, 1.2, 1, 0.9, and 0.8 mm. The pattern was plotted by the AxiDraw and immediately cured in an oven for 30 min at 150 °C. Once the ink cured, we again tested the water resistance of each circle by adding a yellow dye drop to the center of each printed circle. For the speed and resolution tests at each nib size, images of each circle were captured using the MM-840 EFLEX digital microscope (Figure 4a–c). The images were then subsequently analyzed using a MATLAB script (MathWorks, Natick, MA, USA) to determine the inner and outer radius of each circle. The radii were measured at 8 different angles (0°, 45°, 90°, 135°, 180°, 225°, 270°, and 315°) and then used to determine the thickness of the plotted circle.

We then used a 0.1 mm nib to plot a microfluidic device (on laminated Whatman regenerated paper) designed to find the minimum possible channel width that allowed for reproducible flow. The device consisted of a central sample well with eight equally spaced channels of plotted width 200 μm, 300 μm, 350 μm, 400 μm, 450 μm, 500 μm, 600 μm, and 700 μm around the center well (Figure 4d). Once the design was cured in the oven at 150 °C for 30 min, we introduced 10 μL of yellow dye to the center well and observed which channels allowed the dye to flow from the center well to the outer bulbs (Figure S1). Images of each channel were taken from both the front and back of the paper using the MM-840 EFLEX digital microscope (Figure S2). Each channel image was then processed using ImageJ to measure the thickness of the channel at 10 equidistant locations across the length of the channel. Next, each functioning channel was cut in half to reveal the channel’s cross-section. The MM-840 EFLEX digital microscope was again used to capture images of each channel’s cross-section, revealing the channel’s shape within the paper (Figure 4e).

To determine the directional differences in flow rate across Whatman regenerated paper, we designed a modification of the channel size test. The new device again consisted of a central dye well with 8 equal channels of equal width around it. Using a Copic Multiliner SP pen in the AxiDraw pen plotter, we printed the equal channel design three times using a nominal channel width of 400 μm, 550 μm, and 700 μm. Once cured in an oven at 150 °C for 30 min, 10 μL of aqueous yellow food dye was introduced to the central well (Figure 4f). The MM-840 EFLEX digital microscope was used to capture video of the dye flowing through the channels for further time analysis (Figure S3).

### 2.4. Packaging Detection Zones

**Figure 5:**
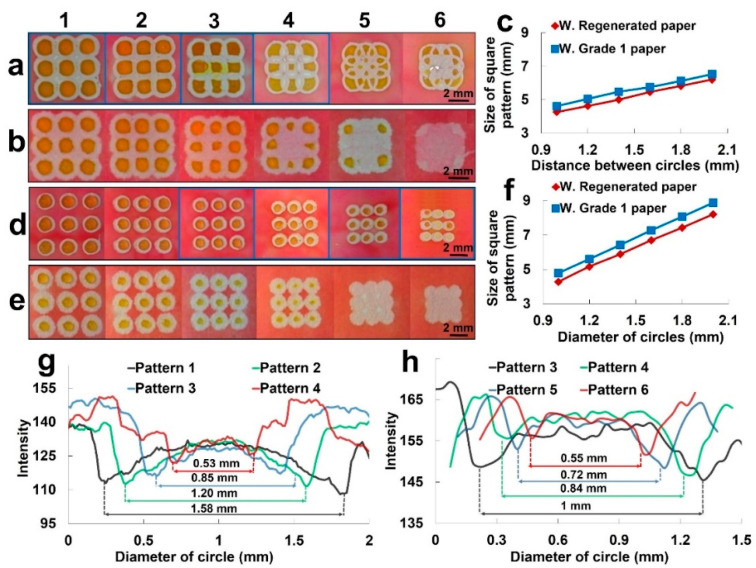
Packing density analysis of closely patterned detection zones. (**a**,**b**) High-resolution packing of detection zones on Whatman regenerated paper (**a**) and Whatman grade 1 paper (**b**). The diameter of circles in each pattern is 2 mm. The center-to-center distance between adjacent circles starts at 2 mm in the first pattern and decreases by 0.2 mm in each pattern. (**c**) Graph of pattern size versus center-to-center distance between adjacent circles in subfigure a and b (*N* = 9 and the error bar shows the standard deviation). (**d**,**e**) High-resolution fabrication of detection zones on Whatman regenerated paper (**d**) and Whatman grade 1 paper (**e**). The diameter of circles and center-to-center distance in the first pattern is 2 mm and 3 mm, respectively. The diameter of circles and the center-to-center distance between adjacent circles decrease by 0.2 mm and 0.3 mm in each pattern, respectively (the diameter of circles and the center-to-center distance of pattern 6 are 1 mm and 1.5 mm, respectively). (**f**) Graph of pattern size versus diameter of circles of subfigures d and e (*N* = 9 and the error bar shows the standard deviation). (**g**) Graph of grayscale intensity analysis of food dye in detection zone of pattern 1–4 of subfigure a. (**h**) Graph of grayscale intensity analysis of food dye in detection zone of pattern 3–6 of subfigure d.

We optimized how tightly we can pack the plotted patterns to fabricate microfluidic devices with the maximum number of detection zones per unit of area. In this part, nine circles with diameters of 2 mm were patterned with a 0.1 mm nib size in a 3-by-3 square matrix. Initially, the distance from centroid-to-centroid of neighboring circles was 2 mm in the first square pattern. We gradually decreased the distance from centroid-to-centroid from 2 mm to 1 mm with 0.2 mm intervals (Figure 5a–c). We performed this experiment on both the Whatman regenerated paper and the Whatman grade 1 paper, to demonstrate the effect of paper thickness on patterning performance. Then, color intensity analysis was performed using ImageJ software to show the coffee ring effect and the optimum centroid-to-centroid distance.

In addition, we optimized packaging for different size of detection zones. A 3-by-3 square of patterned circles was again plotted with a 0.1 mm nib size pen; however, in this packaging experiment, both the diameter of circles and the centroid-to-centroid distance of neighbor circles were gradually decreased until we determined the smallest possible circle that can be plotted. The object of this experiment was to characterize and compare both the Whatman regenerated paper and the Whatman grade 1 paper to reach minimum drawable circle sizes in order to increase the number of detection zones per unit of area. In the first pattern, the diameter of each circle was 2 mm and the centroid-to-centroid distance of the neighbor is 1.5 times the diameter, which was 3 mm. Then, using a 0.2 mm interval, the diameter was decreased down to 1 mm and the neighboring distance decreased down, depending on the diameter, to 1.5 mm (Figure 5d,f). In order to understand the coffee ring effect and quantify the working area in a detection zone that can be usable in a real application, the color intensity analysis was performed on square patterns (Figure 5g,h). We used ImageJ image processing software to make measurements of square patterns and for color intensity analysis. After plotting the patterns, we applied yellow dye at the center of each circle and red dye on the rest of paper to show the hydrophobic barriers.

### 2.5. High Resolution 3D Paper Microfluidic Device

**Figure 6:**
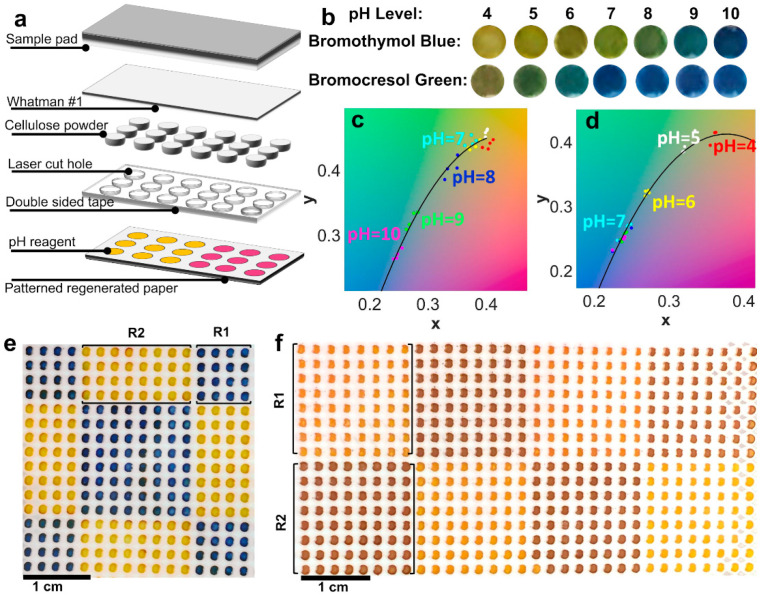
High-resolution and multiplex paper-based microfluidic analytical device. (**a**) Layers and fabrication steps of the high-resolution paper-based microfluidic device. (**b**) Color change of the indicators, i.e., bromothymol blue and bromocresol green, for a range of pH 4 to 10. (**c**,**d**) Calibration curves for determining pH of the solutions by two indicators, bromothymol blue (**c**) and bromocresol green (**d**) using CIExyY color space. (**e**) Images of a device with 256 detection zones after applying the pH 10 sample to the sample pad. Using the technical pen and pen plotter, the detection zones are automatically filled with bromothymol blue (R1) and methyl red (R2) pH indicators with any given arbitrary pattern. (**f**) Photograph of a device with 512 detection zones filled with bromothymol blue (R1) and methyl red (R2) pH indicators, after applying the pH 4 sample to the sample pad.

To show the applicability of the presented method, we plotted a matrix of circles with diameter of 1.6 mm. After patterning the laminated regenerated paper, the pH indicators were spotted in the detection zones using the technical pen and dried in room temperature. The indicators used were bromothymol blue (0.8 mg/mL), bromocresol green (0.8 mg/mL), and methyl red (2.5 mg/mL). To be able to deliver a sample simultaneously to multiple detection zones, we designed a multilayer paper-based device consisting of a laminated Whatman regenerated paper, a layer of double-sided tape, cellulose powder, Whatman grade 1 paper, and Fusion 5 sample pad (Figure 6a). The double-sided tape was cut by a CO2 laser cutter and attached to the laminated regenerated paper which were patterned. To make sure that the sample wicked reproducibly between the layers of the device, the holes of the double-sided tape were filled with cellulose powder, the excess powder was wiped out, and, after peeling the plastic backing of the tape, the cellulose remained in the holes. Then, the Whatman grade 1 paper and sample pad were stacked on the top. To show the ability of the device to quantitatively measure pH levels from 4 to 10, we used two indicators (0.8 mg/mL of bromothymol blue and bromocresol green) (Figure 6b) and obtained calibration curves (Figure 6c,d). To demonstrate the functionality of the proposed method for high-resolution and high-throughput fabrication of multiplex assay paper-based devices, we fabricated devices with 256 and 512 detection zones and tested them with pH 10 and pH 4 samples, respectively (Figure 6e,f).

## 3. Results and Discussion

### 3.1. Increase Pattern Resolution on Whatman Grade 1 Paper

To increase the resolution of the pen plots on Whatman grade 1 paper, a wide Copic marker attached to an Axidraw pen plotter was used to coat the backside of paper (Figure 2a). This decreased the paper’s effective thickness, which made it possible to increase the pen plotting speed/resolution. The pen plotter speed is determined as a percentage of the maximum linear speed, where 30% is equivalent to 55.3 mm/s (Figure S4).

To measure the effects of the wide marker coating on reducing the thickness of the chromatography paper, the marker was plotted for various speeds (Figure 2b,c). We measured the dye penetration thickness at 10 points for each marker speed. A higher dye penetration signaled a larger effective thickness of the chromatography paper. As the drawing speed was increased, the effectiveness of the hydrophobic solution on reducing the thickness of the paper decreased linearly (Figure 2b,c).

After we confirmed the effectiveness of the wide marker drawing to reduce the paper thickness, we investigated how this influenced the resolution of the pen circle plots. At different paper thicknesses, circles were plotted for various speeds and food dye was used to observe any leakage. We found that, at smaller paper thicknesses, the pen was able to draw at higher speeds. These higher speeds decreased the hydrophobic barrier thickness required to avoid leakage. We were able to maximize the resolution of the pen plots with a paper thickness of 50% (Figure 2d,e), resulting in an average barrier thickness of approximately 280 μm (Figure 2d). Figure 2e shows the image of circular pattern with 2 mm diameter on the Whatman grade 1 paper with 50% effective thickness and grayscale intensity analysis of food dye in detection area to evaluate the coffee ring effect of the detection zones.

### 3.2. The Physical Characterization of Paper Substrates

Figure 3a,b show the 45° view SEM image of the laminated Whatman grade 1 paper at 250× and 750× magnification, showing the surface and cross-section of the substrate. We have also imaged the surface of Whatman grade 1 paper at 250× and 750× magnification to characterize the cellulose fiber and pore structure (Figure 3c,d). Figure 3e,f show the 45° view SEM image of the laminated Whatman regenerated paper at 500× and 1500× magnification, showing the surface and cross-section of the substrate. We have also imaged the surface of Whatman regenerated paper at 5000× and 10,000× magnification to characterize the cellulose fibers and pore structure (Figure 3g,h). Comparing the physical features of Whatman regenerated paper (Figure 3g,h) and Whatman grade 1 paper (Figure 3c,d) implies that the cellulose fiber and pore structure of Whatman regenerated paper is around 20 times finer than Whatman grade 1 and the former has higher porosity and homogeneity of pore structure. In addition, the nominal thickness of Whatman regenerated paper is 80 μm, which is more than two times thinner than the Whatman grade 1 paper with 180 μm thickness (Figure 3a,e); that makes the Whatman regenerated paper a better choice of substrate for fabrication of high-resolution paper-based microfleece devices.

### 3.3. The Effect of Nib Size on Resolution

The goal of testing the effect of nib size on resolution was to design the highest resolution channels and reaction areas possible using a Copic Multiliner SP pen in the AxiDraw pen plotter. For the 0.03 mm nib, we found the fastest plotting speed that still created hydrophobic barriers to be 3%. Using the 0.03 mm nib size at 3% speed, we were able to plot eight different reaction area sizes from 0.8 mm to 2.0 mm in radius. Each size was plotted nine times. The smallest reaction area plotted (0.8 mm) had a hydrophobic barrier thickness of 305 ± 86 μm (Figure 4a). For the 0.05 mm nib, the fastest plotting speed that still created an effective hydrophobic barrier was 6%. Using the above circle sizes and repeats, we tested again with the 0.05 mm nib at the optimal 6% speed. The smallest reaction area plotted (0.8 mm) had a barrier thickness of 310 ± 79 μm (Figure 4b). For the 0.1 mm nib, we found we were able to create a hydrophobic barrier at 11% plotting speed. Using the aforementioned circle sizes and repeats, we tested the 0.1 mm nib at 11% plotting speed. The smallest reaction area plotted (0.8 mm) had a barrier thickness of 448 ± 134 μm (Figure 4c). The results showed that an increase in the nib size enabled the setup to plot the hydrophobic barriers at higher plotting speeds. The thicker nibs provided the required amount of the hydrophobic ink in a shorter time; therefore, faster plotting speeds could be used to create an effective hydrophobic barrier. Figure 4a–c showed that as the radius of the reaction area increased from 0.8 mm to 2 mm, the average hydrophobic barrier thickness did not significantly change. This could be attributed to the mechanical consistency in plotting. Although we were able to increase the patterning speed as the nib size increased, the hydrophobic barrier thickness did not remain constant, but increased. This showed that the nib size parameter was dominant over the increase in plotting speed, which led to more ink deposition on the paper and resulted in a thicker hydrophobic barrier.

While the 0.03 mm and 0.05 mm nibs produced very high-resolution results for the reaction area circles, we found that both nibs gradually began to clog as more and more devices were plotted. Over time, the hydrophobic ink dried on the inner surface of the pen, making the nib narrower. This issue was more serious for the 0.03 mm and 0.05 mm nibs and happened much sooner. As they clogged, less ink was deposited onto the paper causing the leakage of the sample through the plotted hydrophobic barriers. Conversely, the 0.1 mm nib did not clog, even after multiple days of testing. For this reason, we decided that the 0.1 mm nib was the most suitable nib for further testing and applications, as it did not clog after repeated use and still achieved a resolution of 448 ± 134 μm.

### 3.4. Resolution of Plotted Channels

In maximizing the channel resolution, we found that the smallest reproducible channel fabricated was the 350 μm-plotted channel (Figure 4d). This channel had a measured width of 150 ± 12.9 μm on the backside of the paper. The front of this channel was completely closed off at the surface. To our knowledge, this is the highest resolution for any paper-based microfluidic flow channel across all known fabrication methods. Once greater than or equal to 450 μm, the measured channel width was shown to linearly increase with the plotted width. The standard error was quite consistent across each channel, indicating that the error came primarily from the inconsistency in the ink wicking horizontally into the channel despite the designed channel width.

When the pen nib releases ink into the laminated Whatman regenerated paper, the hydrophobic ink wicks into the paper vertically and horizontally. Due to this horizontal wicking, the barriers created by the 0.1 mm nib at 11% speed closed into the designed channel width, decreasing the channel width by roughly the thickness of the barrier. In the cases of the 200 μm and 300 μm channels, the ink wicked enough into the channel to completely close it, allowing for no microfluidic flow, as shown in Figure 4d. Additionally, due to the nature of the horizontal and vertical wicking in the paper, the barrier was thicker on the surface of the paper than at the base of the paper. This caused the channel between the two barriers to be wider at the base of the paper than on the surface. In some instances, the channels were completely closed on the surface, yet were still open underneath the surface. Cross-sectional imaging of each channel revealed that below the surface layer of hydrophobic ink (seen on the 350, 400, and 450 channels in Figure 4d), there was, indeed, a channel that allowed for microfluidic flow from the inner well to the outer bulb (Figure 4e and Figure S1). Figure 4e demonstrates how, as the plotted width increased from 350 μm to 700 μm, the channel widened, becoming visible from the surface of the paper.

After performing the channel test, it became evident that the flow rate through the Whatman regenerated paper was not consistent in every direction. In an effort to study the differences in directional flow through the paper, we plotted the channel test again, but with equal channel widths in all eight directions. Analyzing the flow in each direction demonstrated that the directional flow was not consistent; however, the SEM images of Whatman regenerated paper (Figure 3e–h) show high resolution and homogeneity of pore structure in all directions, not supporting the trend that the Whatman regenerated paper allowed better flow in one specific direction than in any other direction. While no direction was found to be flow-dominant, the average time to flow in all eight directions did provide insight into the variance trend of flow time (Figure 4f and Figure S2).

Based on Washburn’s equation, the imbibition speed depends on the characteristics of paper such as average pore diameter, effective surface tension, and the capillary contact angle [2]. Therefore, the imbibition speed remains the same for channels with different widths on the same paper. This equation is not applicable for channels restricted with hydrophobic boundaries. Our results show that the channel width affects the imbibition speed due to the effect of hydrophobic boundaries. This can be explained by the fact that the surface tension at the hydrophobic boundaries is against the flow direction, which slow down the imbibition speeds, especially at narrower channels [41]. There are studies confirming the effect of the hydrophobic barriers on the imbibition dynamics in the channel as well as the correlation between imbibition speed and the channel width [41]. Experimentally, we can see that as the channel width increases, the time to fill decreases, and the standard deviation significantly decreases (Figure 4f). This trend teaches us that the inconsistency in the flow time is dominated by the fabrication, especially at lower channel widths. As the channel width increases, the variation is mainly due to minor flow inconsistencies in the paper itself.

### 3.5. Packing and Resolution of Reaction Areas

Figure 5a,b shows the packing of detection zones on Whatman regenerated paper and Whatman grade 1 paper, respectively. When the distance between the circles decreases, the inner area of the detection zone decreases. The center detection zone in Whatman grade 1 paper disappeared after the third pattern, while it disappeared after the fourth pattern in Whatman regenerated paper. Moreover, the inner area of the detection zones in Whatman grade 1 paper was smaller than in Whatman regenerated paper. Figure 5c shows the size of square patterns versus the center-to-center distance between adjacent circles for both Whatman regenerated and Whatman grade 1 paper. The patterns on Whatman grade 1 paper are bigger than patterns on Whatman regenerated paper by about 6 percent because the ink diffuses more in the former paper than in the latter paper. The grayscale intensity analysis was performed for the highlighted first four patterns in Whatman regenerated paper (Figure 5a). Figure 5g shows the result of this intensity analysis for 2 mm × 0.1 mm horizontal rectangular shape located at the center of the central circles. The intensity is very high on the barrier line because of its grey color. The lowest intensity value shows that the color is darker near the border due to coffee ring effect. This is because the color pigments in the dye collapse at the border of detection zone. Around the center of the detection zone, the intensity increases to around 125 ± 5 because the center of detection zone has a lighter color than the border. This indicates that pattern 4 has 0.53 mm workable area after packaging, while pattern 1 has a 1.58 mm workable area. Figure 5d,e shows the results of packing for different size of detection zones on Whatman regenerated paper and Whatman grade 1 paper, respectively. The diameter of each circle and the center-to-center distance between neighboring circles from 2 and 3 mm decreased to 1 and 1.5 mm, respectively. The detection zones in Whatman grade 1 paper disappeared at a diameter of 1.4 mm (after pattern 3) because the diffused ink filled the detection zones. However, the Whatman regenerated paper had enough space in each detection zone when the diameter reached 1 mm in the last pattern. The size of the last pattern was around 4.6 mm by 4.6 mm, and it covered nine detection zones. Figure 5f shows the size of the square patterns versus the diameters of circles across each pattern for both Whatman regenerated paper and Whatman grade 1 paper. The Whatman grade 1 paper has around 6 percent bigger size than Whatman regenerated paper because the Whatman grade 1 paper has a thicker line due to more ink needing to be deposited on the paper to ensure a hydrophobic barrier. Figure 5h shows the grayscale intensity analysis of detection zones on Whatman regenerated paper (highlighted patterns in Figure 5b). The analysis was performed by placing a 1.5 mm × 0.1 mm horizontal rectangle at the center of the detection zone for all four patterns, maintaining the same rectangle size as diameter decreased for consistency. The grayscale intensities for all four patterns start with a high value around 165 on the border. Around the center of the circle, the intensity drops significantly due to the coffee ring effect, which occurs at the outer edge of the circles. Then, moving closer to the center, the intensity increases and stabilizes around 160 ± 5 at the center of detection zone. Due to the coffee ring’s effects, the intensity reaches a minimum value at the outer edge of the circle. The intensity then gradually rises again towards the center of the circle and stabilizes in the workable detection zone. These intensity shifts can be seen even in the smallest circle of 1 mm in the last pattern. The 1 mm circle has a useable area of around 0.55 mm in diameter after the coffee ring effects.

### 3.6. Colorimetric Quantification

The smartphone’s complementary metal–oxide–semiconductor (CMOS) sensor stores the image of the colorimetric assays in non-linearized sRGB (red, green, blue) values. Simple RGB analysis of test assays does not produce distinguishable concentration levels of the sample. Therefore, after linearization of the raw data, the RGB intensity-values were converted to *xyY* values and plotted on the CIE 1931 color space. Linearization of sRGB values was achieved through the following equations:

$$R_{linear}={\left(\frac{0.055+R_{srgb}}{1.055}\right)}^{2.4}$$

$$G_{linear}={\left(\frac{0.055+G_{srgb}}{1.055}\right)}^{2.4}$$

$$B_{linear}={\left(\frac{0.055+B_{srgb}}{1.055}\right)}^{2.4}$$

The linearized RGB values, falling between 0 and 255 on the color scale, were converted to the corresponding CIE 1931 *XYZ* color space and then to the CIE 1931 *xyY* color space. The following equations were used for such conversions from the linearized RGB values:

$$\left[\begin{array}{c} X\\ Y\\ Z \end{array}\right]=\left[\begin{array}{ccc} 0.4124 & 0.3576 & 0.1805\\ 0.2126 & 0.7152 & 0.0722\\ 0.0193 & 0.1192 & 0.9505 \end{array}\right]\left[\;\begin{array}{c} R_{linear}\\ G_{linear}\\ B_{linear} \end{array}\right]$$

The *X*, *Y*, *Z* tristimulus values were then converted to *xy* chromaticity values, where the *Y* parameter represents luminance of the color, using the following equations:

$$x=\frac{X}{X+Y+Z}$$

$$y=\frac{Y}{X+Y+Z}$$

The *xyY* parameters define the 2D chromaticity diagram in the CIE 1931 color space. By creating a calibration curve from the standard analyte solutions, the chromaticity diagram can be used to predict the outcome of color mixtures, aiding in the identification and quantification of complicated colorimetric assays.

### 3.7. Multiplex 3D Paper-Based Microfluidic Devices for pH Assay

We fabricated devices with high-resolution patterns and a high number of detection zones by stacking layers of laminated regenerated paper, double-sided tape, and chromatography paper (Figure 6a). We showed that by using two pH indicators (0.8 mg/mL bromothymol blue and 0.8 mg/mL bromocresol green) jointly, the pH levels from 4 to 10 can be determined with high precision (Figure 6b–d). Bromothymol blue works well for determining the pH of neutral and basic solutions and bromocresol green is suitable for acidic and neutral pH. The equation of calibration curves and *R*-squared values obtained using bromothymol blue and bromocresol green are, respectively:

$$pH_{BB}=240.31x-144.25y-415.96x^{2}+194.55y^{2}-0.74;\;R^{2}=0.96$$

$$pH_{BG}=667.02x-348.19y-1.025\times 10^{3}x^{2}+416.21y^{2}-30.77;\;R^{2}=0.9$$

Moreover, we demonstrated the capability of our approach to fabricate multiplex devices with 256 and 512 detection zones that could have any desired reagent pattern (Figure 6e,f). On the other hand, by increasing the number of detection zones, the risk of cross-contamination (especially for the base samples which generate darker color) increases. To prevent this issue, optimizing the amount of the sample and increasing the number of layers of the device may be helpful. Two pH indicators are used in each device; the color of methyl red (bromothymol blue) indicator changes from red (yellow) to yellow (blue) when the pH of the sample changes from acidic to basic pH. Once the sample is introduced to the top layer of the device (sample pad), the color of the indicators changes. Using two indicators in every single device increases the precision of the device in determining the pH of the sample.

A device with a matrix of 100 circles can be patterned in 102 s. The fabrication time increases when we increase the number of detection zones. In addition to the high speed and high resolution of the presented method, this method leads to the cost-effective devices. The approximate fabrication cost of a device with 100 detection zones is about $1.

## 4. Conclusions

Here, we have demonstrated the use of a desktop pen plotter in combination with a high-resolution technical pen and a uniformly porous regenerated cellulose paper. This approach pushes the limits of spatial assay resolution and offers a low-cost, high-resolution, and high-throughput method for fabrication of miniaturized paper-based devices. The pen plotting approach presented here has employed low-cost technical pen and hydrophobic ink without any cleaning or clogging issue and has channel resolution of 150 ± 12 µm. To create a functional multiplex device, this approach is capable of fabricating hydrophobic barriers of detection zones and automatically depositing the reagents with any given arbitrary pattern. To show a practical application of the presented approach, we fabricated a 3D paper-based microfluidic device with hundreds of detection zones for conducting pH colorimetric assays. Here, we used two different reagents, but multiple reagents with any given pattern could be deposited in the detection zones, making this method a strong fit to automatically fabricate miniaturized devices with multiplex assays.

## Supplementary Materials

The following are available online at https://www.mdpi.com/2072-666X/11/6/611/s1, Figure S1: Flow test of various channel sizes, Figure S2: The front and back of the 500 μm channel from the 250–700 μm channel flow testing, Figure S3: Images of the equal channel flow testing, Figure S4: Plotting speed in units of mm/s vs percentage; linear velocity (mm/s) was calculated by measuring the time needed for plotting a 25 cm line, Table S1: Comparison among analog fabrication methods, Table S2: Comparison among digital fabrication methods.
