# Supplementary material for: Pushing the Limits of Spatial Assay Resolution for Paper-Based Microfluidics Using Low-Cost and High-Throughput Pen Plotter Approach
Source: Micromachines (Basel). 2020 Jun 24;11(6):611. doi: 10.3390/mi11060611 (PMC7344671; doi:10.3390/mi11060611)
Supplement: Supplementary file 1 [file micromachines-11-00611-s001.pdf]

Supplementary Materials: Pushing the Limits of Spatial Assay Resolution for Paper-Based Microfluidics Using Low-Cost and High-Throughput Pen Plotter Approach

Reza Amin, Fariba Ghaderinezhad, Caleb Bridge, Mikail Temirel, Scott Jones, Panteha Toloueinia and Savas Tasoglu

Calibration of the Pen Plotter

Prior to using the AxiDraw desktop pen plotter to plot tests, it was first important to calibrate the plotter to ensure uniformity of tests. We calibrated the plotter by setting the 'up position height' to 57%, placing glass plate underneath the penholder and placing the Copic Multiliner SP pen in the holder so the tip rests gently on the glass plate. Next, we removed the glass plate and set the 'down position height' to be 53%, lowering the pen tip just enough to make proper contact with the desired surface. Once the pen was appropriately in the holder, the added delay after lowering the pen was set correctly so that the pen did not sit statically on the below Whatman Paper (No. 1) or begin to move horizontally before the pen contacted the paper. Using slow motion imaging, the delay was adjusted to +25 ms. At this value, the pen was found to begin drawing the desired pattern just as it touched down to the surface. It is important to note that in between each plotting, the pen tip was placed in an inkwell to ensure the tip did not dry out.

Table S1. Comparison among analog fabrication methods.

Fabrication Technique	Resolution (μm)		High-Through-Put	Equipment	Cost	Advantages	Limitations
	Barriers	Channels					
**Photo-lithography [1]**	186 ± 13	184 ± 12	No	-Printer<br>-UV light	Low	-Can be cured in sunlight, reducing necessary equipment<br>-Rapid, inexpensive, and does not require clean room	-Several steps involved in the preparation and production of devices<br>- Hydrophilic channels are exposed to polymers or solvents<br>-Automation requires expensive machinery
**Screen printing [2]**	380 ± 40	671 ± 50	Yes	-Screen<br>-Polystyrene (polymer solution)	Low	-Simple fabrication<br>-No pre-modification: Hydrophilic channels not exposed to polymers or solvents<br>-Potential for mass production	-Chemicals necessitate fume hood access<br>-Automation requires expensive screen printing machines
**Flexographic printing**	N/A	500 ± 30 [3]	Yes	-Commercial printing equipment [4]	High [5]	-No pre-modification: Hydrophilic channels not exposed to polymers or solvents<br>-Allows direct roll-to-roll production in existing printing houses [6]	-Requires frequent cleaning to avoid contamination<br>-Complex reagents and templates [5]<br>-Requires different printing plates [6]
**Stamping [7]**	357 ± 28	428 ± 21	No	-Flash stamp machine<br>-Inkjet printer (for masking)<br>-Flash foam	Low	-No pre-modification: Hydrophilic channels not exposed to polymers or solvents	-Requires specialized flash stamp machine<br>-Multi-step preparation and production<br>-Automation requires expensive machinery
**Lacquer spraying [8]**	N/A	N/A	No	-Patterned iron mask<br>-Magnetic plate<br>-Acrylic lacquer (spray)	Low	-Simple and fast method<br>-No pre-modification: Hydrophilic channels not exposed to polymers or solvents	-Manual process; would require expensive machinery for automation<br>-Iron mask must be laser cut
**Plasma treatment [9]**	N/A	1500	No	-Oven<br>-Cut-patterned Metal masks<br>-Vacuum plasma reactor	High	-very high hydrophobicity of barrier and hydrophilicity of channels	-Requires specialized equipment<br>-Automation requires expensive machinery<br>-Hydrophilic channels are exposed to organic solvents

Table S2. Comparison among digital fabrication methods.

Fabrication Technique	Resolution (μm)		High-Through-Put	Equipment	Cost	Advantages	Limitations
	Barriers	Channels					
**Wax printing**	468 ± 28	228 ± 30 [10]	Yes	-Printer<br>-Hot plate [4]	Low	-Simple and fast [5]<br>-No pre-modification: Hydrophilic channels not exposed to polymers or solvents [11]	-Multi-step processes [4]<br>-Not resistant to high temperature [5]<br>-Requires an extra heating step [6]
**Inkjet printing**	200 [12]	272 ± 19 [12]	Yes	-Modified inkjet printer [4]	Low [5]	-Reagents can be inkjet-printed into the test zones using the printer [11]<br>-Inexpensive inkjet printers<br>-Print high resolution and conductive patterns [6]	-Multiple printing steps [4]<br>-Needing an improved inkjet printer [5]<br>-Print head clogging issue
**Paper cutting and shaping [13,14]**	N/A	1000	No	-X-Y knife plotter<br>-Thermal bonding lamination film (optional – provides rigidity)	Low	-Very simple, fast fabrication	-Resulting devices are very delicate<br>-Handling requires high precision<br>-Automation requires expensive machinery
**Inkjet etching [15]**	N/A	400 ± 40	No	-Piezo-driven inkjet printer<br>-Micro-sized dispenser print head	Low	-High resolution channels (for inkjet methods)<br>-Inexpensive printer equipment	-Requires preparation of paper before printing (multi-step process)<br>-Hydrophilic channels are exposed to organic solvents
**Laser etching [16]**	60	80–250	No	-Carbon dioxide laser	Low	-No consumables necessary for basic microfluidic capabilities<br>-Fast operation time	-Requires wax-like coated papers<br>-Multi-step fabrication (requires the addition of silica nanoparticles after laser etching)
**Our high-resolution pen plotting method**	448 ± 134	150 ± 12	Yes	-Pen plotter<br>-Oven/hot plate	Low	-Single-step integrated fabrication<br>-Reagents can be deposited into the test zones using technical pens<br>-No clogging issue<br>-No pre-modification: Hydrophilic channels not exposed to polymers or solvents<br>-low-cost ink and plotter	-Requires an extra ink curing step by heating

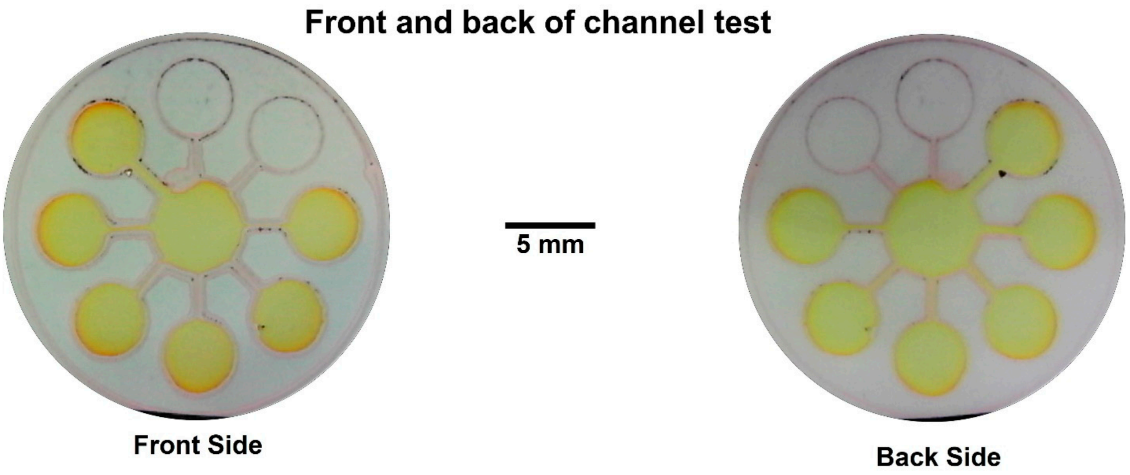

Figure S1. Flow test of various channel sizes. It shows the varied channel flow test from the front and back of the paper. For the front side image, the nominal channel widths, starting at the top and rotating clockwise, are 250 μm, 300 μm, 350 μm, 400 μm, 450 μm, 500 μm, 600 μm, and 700 μm. For the backside image, the nominal channel widths, starting at the top and rotating counterclockwise are 250 μm, 300 μm, 350 μm, 400 μm, 450 μm, 500 μm, 600 μm, and 700 μm. Note that the 250 μm and 300 μm channels were closed completely by the hydrophobic ink and therefore, did not allow the dye to flow.

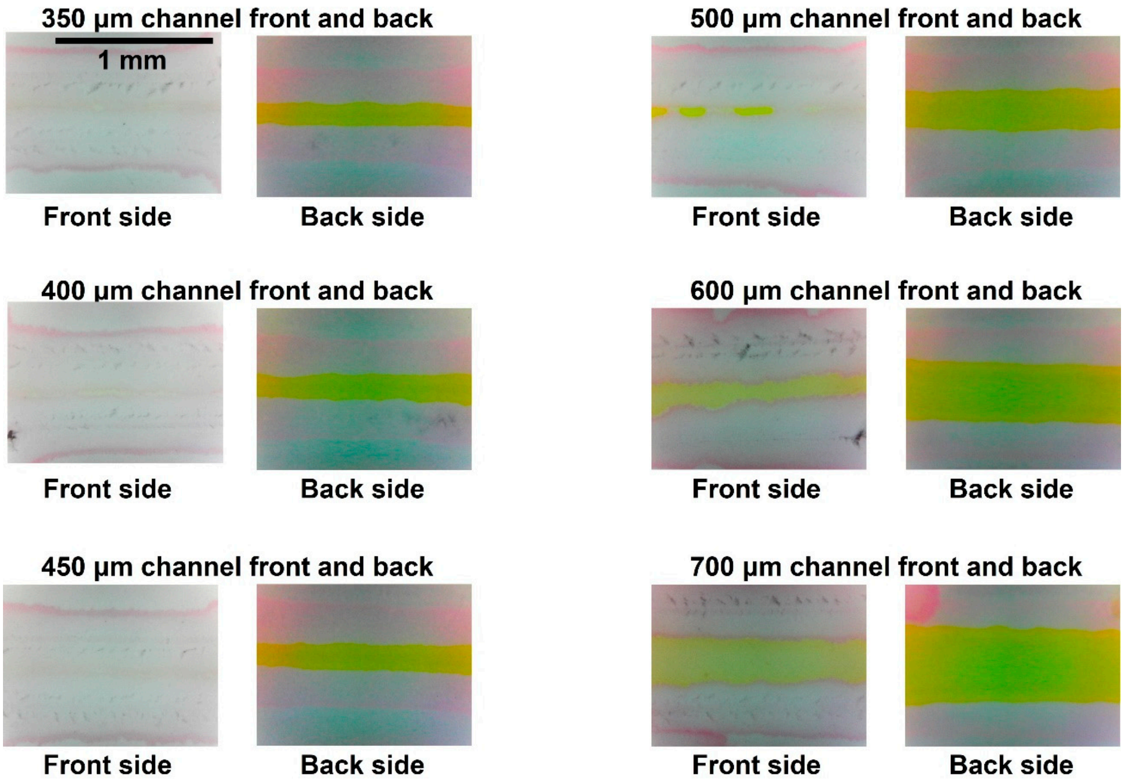

Figure S2. The front and back of the 500 μm channel from the 250–700 μm channel flow testing. The front of the image shows that the channel is mostly closed off by the hydrophobic ink, only allowing the yellow dye to appear slightly; however, the back of the channel is much wider, allowing for flow of the dye. The cross sectional images in Fig. 3e show the same channel geometry. Each back side image was color altered using ImageJ to clearly show the channel edges.

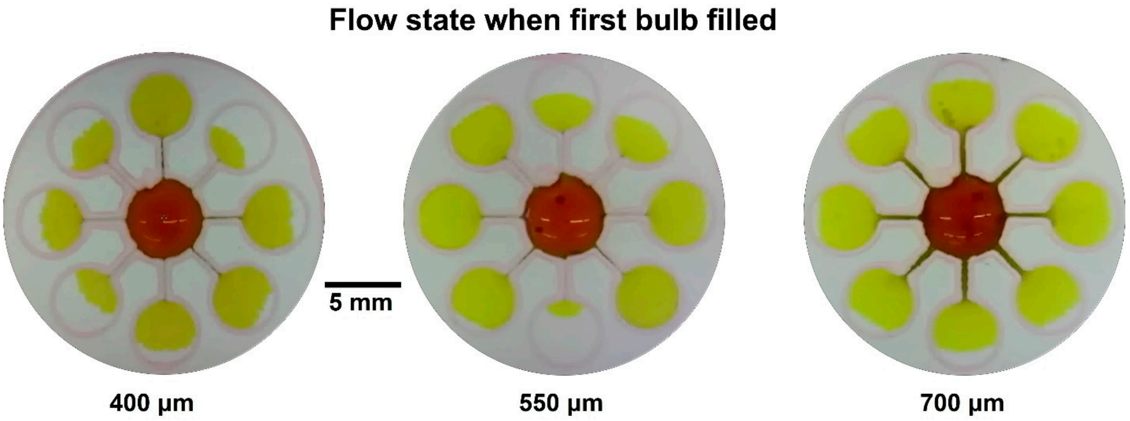

Figure S3. Images of the equal channel flow testing. Each image was captured the instant the first bulb was fully filled. This visually demonstrates how varied the time to fill was for the nominal channel sizes of 400 µm, 550 µm, and 700 µm. The 400 µm channel test is seen to have a wide variance in flow time. Looking to the 700 µm channel, almost all bulbs have filled up at the same time the first bulb fills. This demonstrates that increasing the fabricated channel size decreases that standard deviation of the time for each bulb to fill completely with dye.

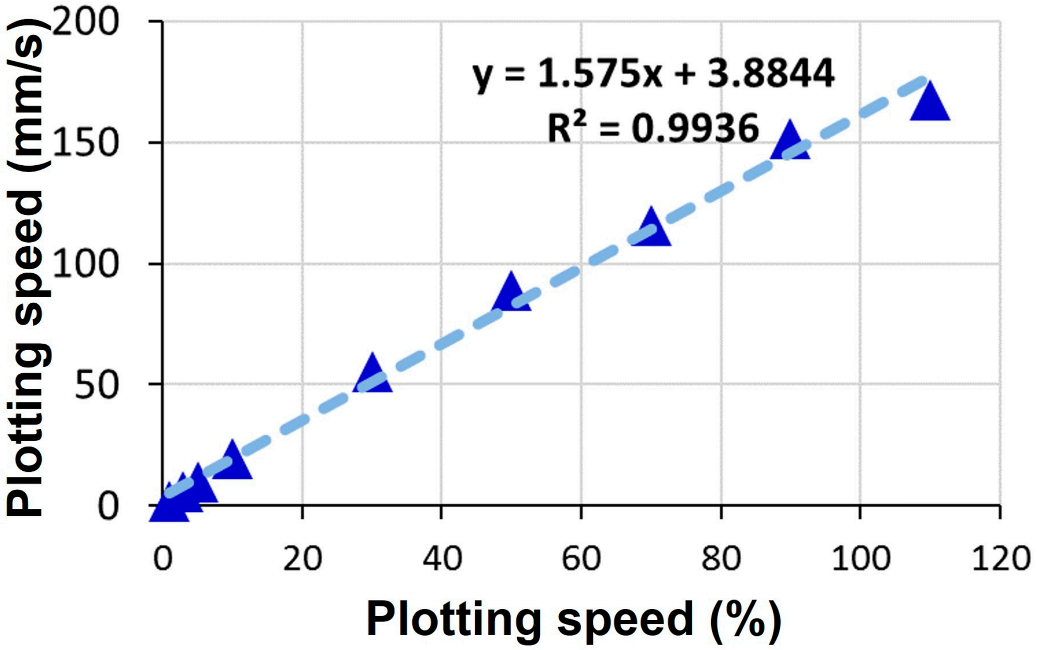

Figure S4. Plotting speed in units of mm/s vs percentage; linear velocity (mm/s) was calculated by measuring the time needed for plotting a 25 cm line.

Reference

1. Martinez, A.W.; Phillips, S.T.; Wiley, B.J.; Gupta, M.; Whitesides, G.M. FLASH: A rapid method for prototyping paper-based microfluidic devices. *Lab a Chip* **2008**, *8*, 2146–50, doi:10.1039/b811135a.
2. Sameenoi, Y.; Na Nongkai, P.; Nouanthavong, S.; Henry, C.S.; Nacapricha, D. One-step polymer screen-printing for microfluidic paper-based analytical device (?PAD) fabrication. *Anal.* **2014**, *139*, 6580–6588, doi:10.1039/c4an01624f.
3. Olkkonen, J.T.; Lehtinen, K.; Erho, T. Flexographically Printed Fluidic Structures in Paper. *Anal. Chem.* **2010**, *82*, 10246–10250, doi:10.1021/ac1027066.
4. Yetisen, A.K.; Akram, M.S.; Lowe, C.R. Paper-based microfluidic point-of-care diagnostic devices. *Lab a Chip* **2013**, *13*, 2210, doi:10.1039/c3lc50169h.
5. He, Y.; Wu, Y.; Fu, J.-Z. Fabrication of paper-based microfluidic analysis devices: a review. *RSC Adv.* **2015**,

5, 78109–78127, doi:10.1039/C5RA09188H.
6. Xia, Y.; Si, J.; Li, Z. Fabrication techniques for microfluidic paper-based analytical devices and their applications for biological testing: A review. *Biosens. Bioelectron.* **2016**, *77*, 774–789, doi:10.1016/j.bios.2015.10.032.
7. He, Y.; Wu, Y.; Xiao, X.; Fu, J.; Xue, G. A low-cost and rapid microfluidic paper-based analytical device fabrication method: flash foam stamp lithography. *RSC Adv.* **2014**, *4*, 63860–63865, doi:10.1039/c4ra11150h.
8. Nurak, T.; Praphairaksit, N.; Chailapakul, O. Fabrication of paper-based devices by lacquer spraying method for the determination of nickel (II) ion in waste water. *Talanta* **2013**, *114*, 291–296, doi:10.1016/j.talanta.2013.05.037.
9. Li, X.; Tian, J.; Nguyen, T.; Shen, W. Paper-Based Microfluidic Devices by Plasma Treatment. *Anal. Chem.* **2008**, *80*, 9131–9134, doi:10.1021/ac801729t.
10. Tenda, K.; Ota, R.; Yamada, K.; Henares, T.G.; Suzuki, K.; Citterio, D. High-Resolution Microfluidic Paper-Based Analytical Devices for Sub-Microliter Sample Analysis. *Micromachines* **2016**, *7*, 80, doi:10.3390/mi7050080.
11. Martinez, A.W.; Phillips, S.T.; Whitesides, G.M.; Carrilho, E. Diagnostics for the Developing World: Microfluidic Paper-Based Analytical Devices. *Anal. Chem.* **2010**, *82*, 3–10, doi:10.1021/ac9013989.
12. Maejima, K.; Tomikawa, S.; Citterio, D.; Suzuki, K. Inkjet printing: an integrated and green chemical approach to microfluidic paper-based analytical devices. *RSC Adv.* **2013**, *3*, 9258, doi:10.1039/c3ra40828k.
13. Fenton, E.M.; Mascarenas, M.R.; LópezG.P.; Sibbett, S.S. Multiplex Lateral-Flow Test Strips Fabricated by Two-Dimensional Shaping. *ACS Appl. Mater. Interfaces* **2008**, *1*, 124–129, doi:10.1021/am800043z.
14. Cassano, C.L.; Fan, Z.H.; Fan, Z.H. Laminated paper-based analytical devices (LPAD): fabrication, characterization, and assays. *Microfluid. Nanofluidics* **2013**, *15*, 173–181, doi:10.1007/s10404-013-1140-x.
15. Abe, K.; Suzuki, K.; Citterio, D. Inkjet-Printed Microfluidic Multianalyte Chemical Sensing Paper. *Anal. Chem.* **2008**, *80*, 6928–6934, doi:10.1021/ac800604v.
16. Chan, H.N.; Chen, Y.; Shu, Y.; Chen, Y.; Tian, Q.; Wu, H. Direct, one-step molding of 3D-printed structures for convenient fabrication of truly 3D PDMS microfluidic chips. *Microfluid. Nanofluidics* **2015**, *19*, 9–18, doi:10.1007/s10404-014-1542-4.
